# Bioselectivity of silk protein-based materials and their bio-inspired applications

**DOI:** 10.3762/bjnano.13.81

**Published:** 2022-09-08

**Authors:** Hendrik Bargel, Vanessa T Trossmann, Christoph Sommer, Thomas Scheibel

**Affiliations:** 1 Department of Biomaterials, University of Bayreuth, Prof.-Rüdiger-Bormann-Str. 1, 95447 Bayreuth, Germany; 2 Bayreuth Center of Material Science and Engineering (BayMat), University of Bayreuth, Universitätsstr. 30, 95440 Bayreuth, Germany; 3 Bavarian Polymer Institute (BPI), University of Bayreuth, Universitätsstr. 30, 95440 Bayreuth, Germany; 4 Bayreuth Center of Colloids and Interfaces (BZKG), University of Bayreuth, Universitätsstr. 30, 95440 Bayreuth, Germany; 5 Bayreuth Center for Molecular Biosciences (BZMB), University of Bayreuth, Universitätsstr. 30, 95440 Bayreuth, Germany

**Keywords:** antifouling, bacteriostatic, biofouling, bioselective cell adhesion, spider silk protein

## Abstract

Adhesion to material surfaces is crucial for almost all organisms regarding subsequent biological responses. Mammalian cell attachment to a surrounding biological matrix is essential for maintaining their survival and function concerning tissue formation. Conversely, the adhesion and presence of microbes interferes with important multicellular processes of tissue development. Therefore, tailoring bioselective, biologically active, and multifunctional materials for biomedical applications is a modern focus of biomaterial research. Engineering biomaterials that stimulate and interact with cell receptors to support binding and subsequent physiological responses of multicellular systems attracted much interest in the last years. Further to this, the increasing threat of multidrug resistance of pathogens against antibiotics to human health urgently requires new material concepts for preventing microbial infestation and biofilm formation. Thus, materials exhibiting microbial repellence or antimicrobial behaviour to reduce inflammation, while selectively enhancing regeneration in host tissues are of utmost interest. In this context, protein-based materials are interesting candidates due to their natural origin, biological activity, and structural properties. Silk materials, in particular those made of spider silk proteins and their recombinant counterparts, are characterized by extraordinary properties including excellent biocompatibility, slow biodegradation, low immunogenicity, and non-toxicity, making them ideally suited for tissue engineering and biomedical applications. Furthermore, recombinant production technologies allow for application-specific modification to develop adjustable, bioactive materials. The present review focusses on biological processes and surface interactions involved in the bioselective adhesion of mammalian cells and repellence of microbes on protein-based material surfaces. In addition, it highlights the importance of materials made of recombinant spider silk proteins, focussing on the progress regarding bioselectivity.

## Review

### 1 Introduction

#### 1.1 Bioadhesive protein surfaces

Biological adhesion is important for all organisms such as plants, animals, bacteria, and fungi, covering a wide range of biological aspects including reproduction, growth and settlement, dynamic attachment during locomotion, self-healing, protection against as well as attachment of microbes, and prey hunting [1–6]. A variety of different adhesion mechanisms exist to manage the different challenges of interfacial adhesion. In turn, natural biological adhesives are complex materials that have evolved to meet the various functional demands. Bioadhesion can be found in the range of the micro- to the macroscale [7]. Adhesion principles include contact mechanical principles such as capillary interactions, viscous forces, non-covalent interactions (e.g., van der Waals forces), and adhesive chemistry of biopolymers (various types of glues) [5]. On the level of tissues, multiple cell types work together to perform complex tasks, based on their hierarchical arrangement governing the exchange of information between different cell types. To achieve proper organization and communication, cells produce a large number of adhesion molecules that mediate cell contact phenomena based on receptor–ligand interactions [8]. The ability of mammalian cells to adhere to other cells, tissues, or the extracellular matrix (ECM) via specific molecular interactions plays a critical role in many biological processes including embryogenesis, development of neuronal tissue, hemostasis, immune response, and inflammation [9]. The adhesive interactions of cells between each other and with ECM proteins (often of specific molecular nature), have important key functions in embryonic and organ development, the maintenance of vascular and epithelial integrity, and host defense [10].

The so-called cellular adhesion molecules (CAMs) can be generally divided into five discrete groups that include cadherins, selectins, members of the immunoglobulin superfamily (IgSF), integrins, and others such as mucins. Moreover, certain enzymes such as the vascular adhesion protein 1 (VAP-1) are known to be involved in cell adhesion [11–12]. The distinction of CAM subcategories reflects both structural differences as well as binding to different ligands. Cadherins, selectins, and members of the IgSF are known to mediate cell–cell adhesion, while integrins typically bind to extracellular matrix proteins [12]. As a special case, immune cell integrins also bind to ligands both soluble and on other cells. Additional classification of the CAMs relates to their ligands and type of adhesion. Selectins bind to carbohydrates based on calcium-dependent regulation, cadherins form mostly homotypic contacts also in a calcium-regulated manner, while the IgSF subfamily of nectins is able to form both homotypic and heterotypic interactions [10]. Comprehensive overviews of molecular structure and adhesion mechanisms of various CAM can be found in dedicated reviews [9–10,12–13].

Cadherins are associated with cell–cell adhesive interactions in solid tissues and are involved in processes such as embryonic development, formation of the epithelial layers of the skin and intestine, and axonal formation in the nervous system [9]. The structural integrity of cadherin molecules is stabilized by calcium ions, and their essential role in proper adhesion contacts is reflected in the abbreviated protein family name “**ca**lcium-**d**ependent ad**her**ent prote**ins**” [14]. The N-terminal domain of cadherin dimers on one cell is responsible for mediating homotypic binding contacts with their homologues on an adjacent cell in an antiparallel manner. This region also defines binding specificity [15]. Some cadherin receptors are also able to bind to different cadherins. Classic cadherin function is involved in key intracellular structures, such as in adherens junctions (zona adherens and adhesion junctions) and protein complexes composed of E-type cadherins tethered to the actin cytoskeleton through the linker protein group of catenins [15]. One example is cardiac muscle, in which β-catenin localizes to adherens junctions, which are critical for electrical and mechanical coupling between adjacent cardiomyocytes [9].

The immunoglobulin superfamily is one of the largest and most diverse protein families. They are characterized by the presence of at least one immunoglobulin or immunoglobulin-like domain. Most members are type-I transmembrane proteins with an extracellular Ig domain, transmembrane domain, and a cytoplasmic tail [16]. IgSF proteins play a critical role in the development of the nervous system, in embryonic development, and in immune and inflammatory responses [9]. Particularly in the immune system, IgSF members play a critical role in cellular adhesion, and well-known examples include major histocompatibility complex (MHC) class I and II molecules, proteins of the T cell receptor (TCR) complex, intercellular adhesion molecules (ICAMs), and vascular cell adhesion molecules (VCAMs) [17]. Mucosal addressing cell adhesion molecule-1 (MAdCAM-1) and activated leukocyte cell adhesion molecules (ALCAMs) also belong to this family of adhesion receptors and are important in leukocyte trafficking events [10]. These proteins serve as ligands for integrins on the endothelial cells. IgSF molecules are divided into several subfamilies, including nectins and selectins. Whereas nectins mediate cell–cell adhesion in various tissues including endothelium, epithelium, and neural tissue [12], selectins are single-chain transmembrane glycoproteins that mediate calcium-dependent binding to sugar moieties [10]. Their most prominent function is associated with the initial stage of the rolling cell adhesion cascade in which selectin binding enables rolling and cell arrest [18].

Integrins are large heterodimeric glycoproteins constituting a diverse family of adhesion molecules. They play a critical role in important biological functions, including embryogenesis, maintenance of tissue integrity, immune response, and inflammation. Integrins consist of two subunits, α- and β- chains, spanning the cell membrane and forming the receptor in the plasma membrane, characterized by noncovalent interactions [10]. Integrins bind to a large variety of ligands, and the majority belong to two categories. The first category are cell surface molecules of the immunoglobulin supergene family, for example, ICAMs and vascular cell adhesion molecule 1 (VCAM-1), and the second category includes larger-sized ECM protein components such as fibronectin, fibrinogen, vitronectin, and complement component iC3b [13]. Divalent cations, such as Mg^2+^ and Ca^2+^, are involved in ligand binding. Individual integrins can bind more than one ligand, and vice versa. However, the cellular environment may influence ligand specificity within a particular cell interaction [9]. Generally, integrin-mediated interactions involve a complex series of events including activation, ligand binding, reorganization of the cytoskeleton, and adhesion [13]. In this context, integrin activation is regulated in response to signal cascades inside the cell known as an “inside-out” process initiated by other cell surface receptors, such as chemokine receptors and selectins. The subsequent interaction with cytoplasmic factors leads to a conformational switch in the extracellular integrin binding region from a non-adhesive (inactive) to an adhesive state [19]. Moreover, adhesive interactions also include activation outside the cell (outside-in process) mediated by the cytosolic domains of the integrin α- and β-subunits. The recruitment of either leukocytes or platelets from the blood circulation are examples that require both signalling pathways [9].

Cells in their native tissue environment are surrounded by a unique, complex ECM consisting of a variety of molecules and proteins showing a diversity of assembled morphologies, structures and folding states, which are essential for their assigned functions. Besides structural and mechanical support, the present proteins are direct interaction partners for the cellular receptors mentioned above and contain binding sites for further biologically active molecules, including growth factors, which stimulate several subsequent cellular responses, such as growth or differentiation [20–23]. Prominent examples of ECM proteins are collagens, laminins, fibronectin, and elastin, which contain several short peptide recognition and binding motifs in their amino acid sequence for interaction with specific cellular surface integrins or other types of receptors [23]. Within the ECM, fibronectin is the best-known attachment site for cells by interacting with members of the integrin family of cell surface proteins. However, fibronectin is a multifunctional adhesion molecule, also binding to other protein constituents of the ECM such as fibrin, collagen, and heparin [24]. Thus, fibronectin is involved in several physiological events such as embryonic differentiation, cell morphology, cell migration, and thrombosis [19]. The function of fibronectin as a cell-binding molecule involves its recognition by integrins through short peptide motifs, among them the prominent Arg–Gly–Asp (RGD) and Leu–Asp–Val–Pro (LDVP) sequences [19].

#### 1.2 Applications of bio-inspired selective surface modifications

The increasing knowledge on natural receptor–ligand binding mechanisms led to various approaches in the last years to increase both cellular adhesion on surfaces of biomaterials and biosensors, as well as for therapeutic drug carrier systems against infections, inflammatory and auto-immune diseases, and for anti-tumor treatments [10,25]. One main goal in developing bioactive, bioadhesive, and functional biomaterial scaffolds for tissue engineering, is the enhanced support and regeneration of injured or non-functional tissues or parts thereof.

Apart from the development of sophisticated synthetic polymer systems [26–27], protein-based and bioinspired materials have nowadays an enormous impact [28–29]. Biomaterials based on native collagen, keratin, elastin, silk, and their recombinant, engineered counterparts become increasingly popular in biomedicine and tissue engineering, since they exhibit promising chemical and physical properties, such as bioactivity, structural integrity, and cell stimulation [29–30]. Biomimetic materials modulating specific cellular responses and tissue regeneration have been developed by adjusting and modifying materials using bioactive and bioadhesive molecules, such as full-length ECM proteins or functional peptide fragments thereof. These ligands interact with cell receptors for guiding cellular responses, such as cell proliferation or specific matrix degradation [31–34]. Compared to full-length proteins, short, bioactive peptide sequences show several advantages due to their size, including similar function, controllable, inexpensive, and synthetic production in high amounts, facile processing, simple modification, accessibility, and no animal origin [31,33].

**Figure 1:**
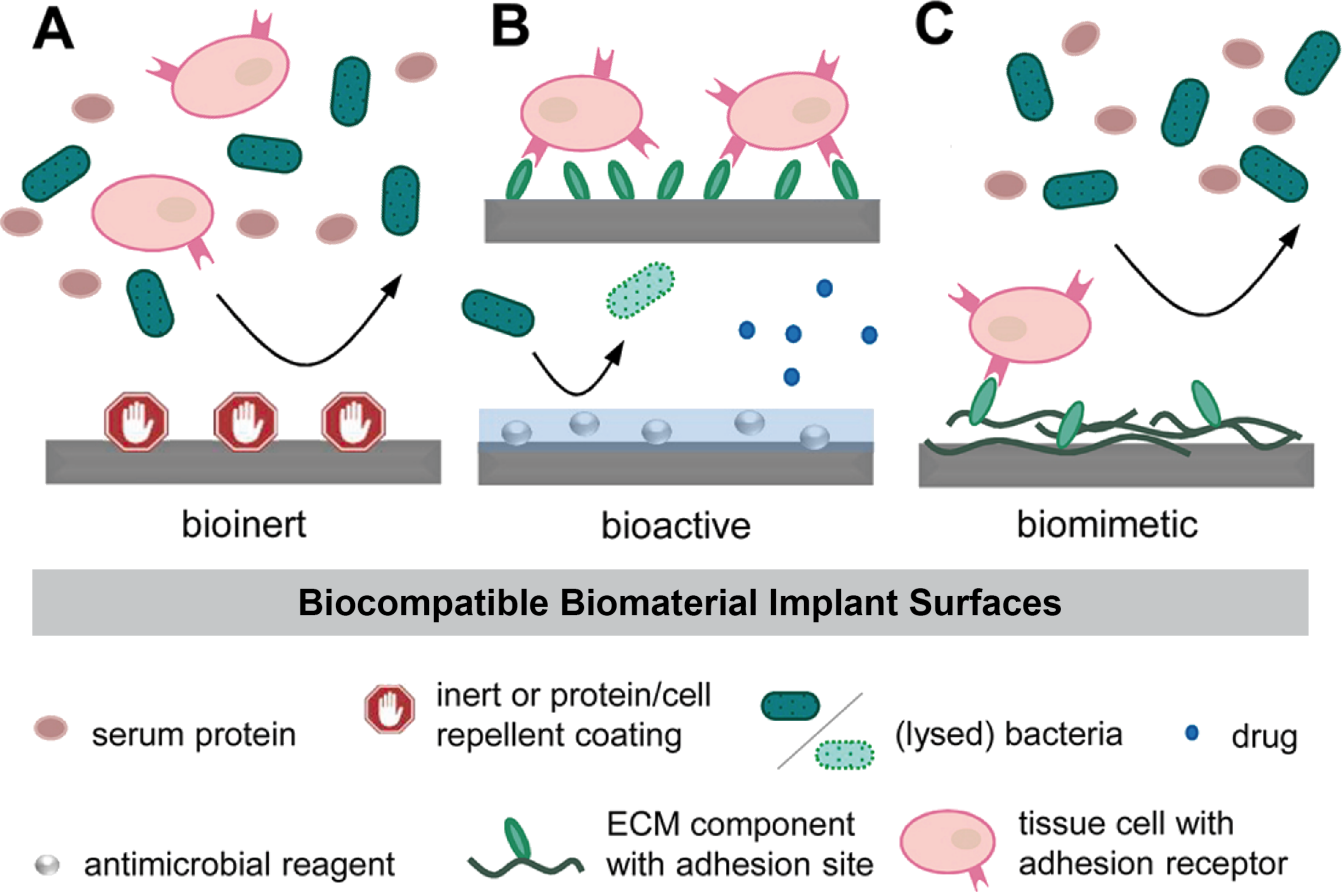
Material-related properties guiding the interactions of proteins, microbes and cells on biomaterial surfaces. (A) Bioinert surfaces hinder the adsorption of proteins, and no interaction and adhesion of cells and microbes is possible. (B) Bioactive surfaces actively stimulate their surrounding by releasing factors or agents to allow for cellular interaction (bioadhesion) or combat against microbes. (C) Biomimetic surfaces are designed to mimic natural ECM composition to guide cellular interactions and tissue regeneration, while repelling proteins and microbes. Figure 1 was adapted from reference [32] (© 2021 S. Spiller et al., published by De Gruyter, distributed under the terms of the Creative Commons Attribution 4.0 International License, https://creativecommons.org/licenses/by/4.0).

Many reviews have summarized biologically active peptide sequences regarding their natural origin, their functions, their cellular effects, the targeted tissues, or the engineered and designed scaffolds [31–36]. The most prominent and important adhesive peptide sequence used for functionalization and increasing the bioadhesiveness of a material is the tripeptide RGD, naturally occurring in various ECM proteins including fibronectin, vitronectin, laminin, and collagen [34–35,37–41]. This frequently used peptide has been shown to increase the bioadhesiveness of biomaterials by interacting with cellular integrin receptors and, thus, allows for interaction and attachment of many different cell types [23,33–34,36,39,42–44]. Some integrin types interact with their ligands by recognizing specific ligand motifs. For instance, α4 integrins recognize the Leu–Asp–Val–Pro (LDVP) motif in fibronectin, the Leu–Asp–Thr–Ser (LDTS) sequence in MAdCAM-1, and Ile–Asp–Ser (IDS) in VCAM-1 [19]. Another example of selective adhesion of cells expressing one type of integrin has been achieved by click chemistry to immobilize peptidomimetics of α5β1- or ανβ3-selective RGD peptides [45]. Two other commonly used adhesive peptides to promote cellular interactions with biomaterials are the pentapeptides IKVAV (Ile–Lys–Val–Ala–Val) and YIGSR (Tyr–Ile–Gly–Ser–Arg), which are naturally present in laminin and are used for increasing bioadhesiveness and cellular interactions with biomaterials [23,33–35,41,46–48]. Functionalization of material surfaces is consistently one important step in tissue engineering, especially in the case of implants, where the interaction of cells with the surface is desired [32,44]. However, in the case of 3D structures, bioactive factors must be present in the bulk material and surround the incorporated cells [31,33–34]. For the usage as implant, biocompatible biomaterial implant surfaces should demonstrate different properties adopted to the needed requirements to allow for a successful incorporation in the human body depending on the aimed application (Figure 1). Therefore, the interactions of proteins, microbes, and cells should be guided by material-related surface properties to create bioinert, bioactive, or biomimetic biomaterials.

#### 1.3 Antiadhesive and anti-fouling protein surfaces

**Figure 2:**
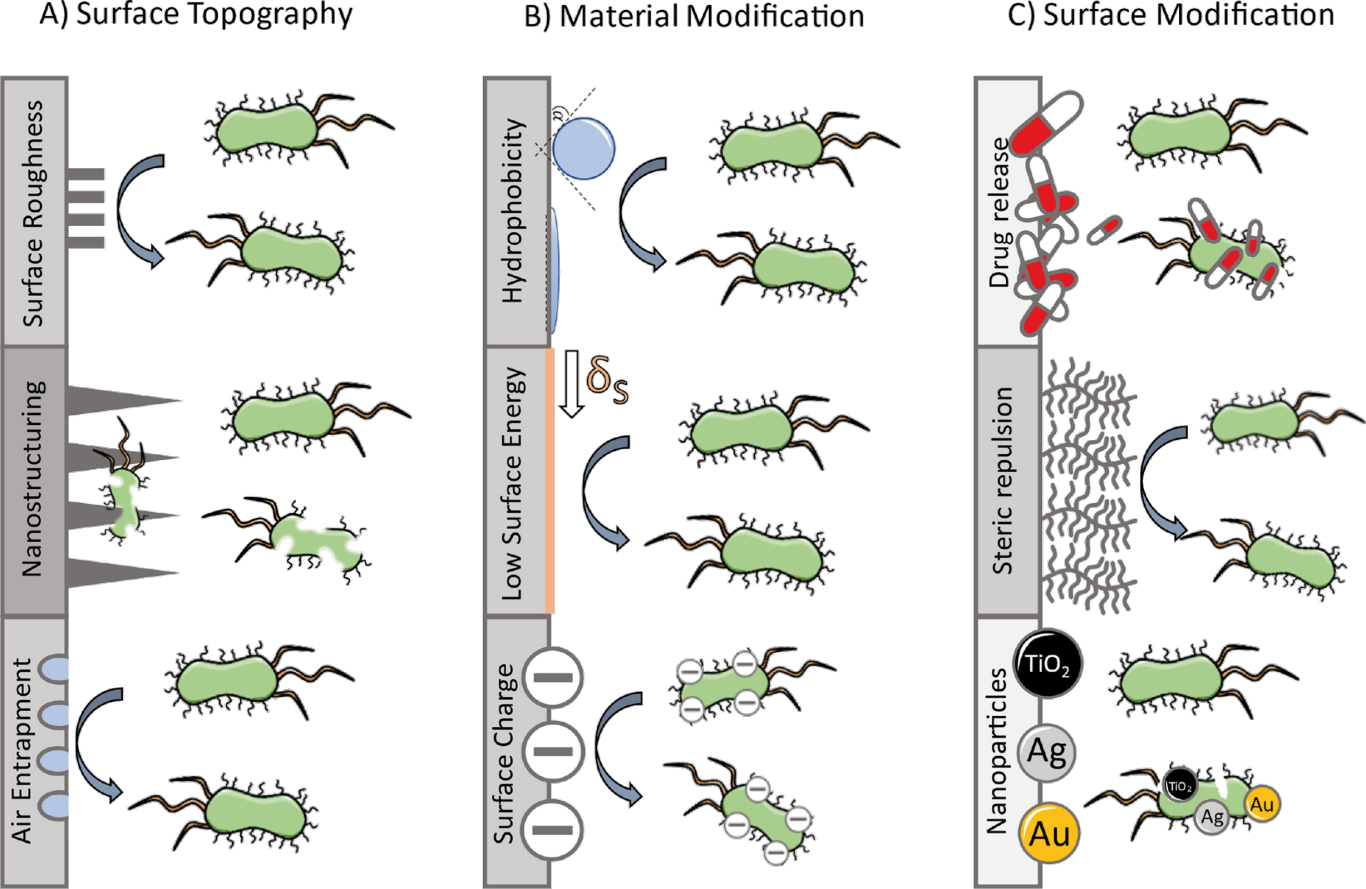
Common antimicrobial approaches regarding the general defense mechanisms (A) surface topography, (B) material modification, and (C) surface modification. Repellent surfaces are displayed in grey, biocide mechanisms in light grey, and contact killing materials in dark grey. Figure 2 was reproduced from reference [50]. (© 2021 D. Sonnleitner et al., published by Elsevier B. V., distributed under the terms of the Creative Commons Attribution-Non-Commercial-NoDerivatives 4.0 International Licence, https://creativecommons.org/licenses/by-nc-nd/4.0/). This content is not subject to CC BY 4.0.

Nature has evolved a diverse portfolio of anti-adhesive, antimicrobial and antifouling methods. Biofouling can be defined as the undesired attachment and growth of life on artificial surfaces [49]. One general strategy to inhibit biofilm formation and microbial colonialization is microbial repellence since it targets a direct inhibition of bacterial adherence on a surface. Various approaches can be distinguished according to their basic defense mechanism. These are (A) surface topography, which disturbs and inhibits the initial adhesion based on morphological features, (B) material modification, where intrinsic chemical and physical properties result in microbe-repellence, and (C) additives and coatings that inhibit initial attachment or directly kill microbes (see Figure 2) [50].

Natural surfaces provide many examples of anti-adhesive topography, including nanostructured pikes on Cicada wings [51], micro-structured and patterned riblets of the shark skin scales [52], hierarchically micro- and nanostructured ultrahydrophobic surfaces with self-cleaning ability, such as Lotus leaves and insect wing analogues [53–55], and the superhydrophobic air-retaining surfaces of *Salvinia* floating fern leaves and of the water bug *Notonecta glauca* [53,56]. Based on such blueprints, bioinspired anti-adhesion and antifouling surfaces have been successfully generated by soft photolithography, micro-molding or nanopatterning techniques [57]. Moreover, the biomimetic application Sharklet^®^ textured similarly to shark skin has not only been reported to be antiadhesive against green algae spores and bacterial cells but to even display antiviral attachment properties [58]. Superhydrophobic surfaces inspired by the Lotus-effect^®^ (>150° contact angle) have been found to diminish bacterial adhesion due to reduced protein surface adsorption [59–61]. Superhydrophobicity relies on the combination of chemical composition, surface structuring on the micro-/nanoscale, and the introduction of low-surface-energy compounds [62]. Various studies demonstrated that the adhesion of *Staphylococcus aureus, Pseudomonas aeruginosa*, and *Escherichia coli* (*E. coli*) bacteria was significantly reduced on superhydrophobic coatings obtained from fluorinated silica colloids, thin film deposition of silicone elastomers or nanoengineered superhydrophobic surfaces of Teflon^®^-coated aluminium [63–65]. Superhydrophobic surfaces have also been reported to be unfavorable for mammalian cell attachment and growth. This may be due to the fact that the proteins in the ECM (such as fibronectin, vitronectin, collagen, and laminin) are adsorbed in a denatured state, and their orientation becomes unsuitable for cell binding [66]. Cells (e.g., osteoblasts) are more likely to attach to more hydrophilic surfaces and exhibit the strongest adhesion at contact angles between 60° and 80° [66].

The contrary material property of highly hydrophilic surfaces also effectively reduces protein and, subsequently, microbial adhesion. A theoretical study analysed the contribution to either adhesion or repellency of steric repulsion, van der Waals attraction, and hydrophobic interaction free energies of surface-bound hydrophilic polyethylene oxide (PEO) polymer [67]. Greater surface density and chain length of terminally attached PEO chains were reported to correlate to a lower van der Waals attractive component. In turn, steric repulsion of the polymer chains against protein molecules increases, while a tightly bound water layer creates a physical and energetic barrier and renders interactions with approaching proteins or bacteria thermodynamically unfavorable [50]. The concept of steric repulsion based on a hydration layer through hydrogen bonding and/or ionic solvation has been reported for various materials such as poly(*N*-isopropylacrylamide) (PNIPAAm) [68–69], dextrans [69], poly(ethyleneimine) (PEI) [70–71], and chitosan [72].

Concerning proteins, several studies have reported different molecules to generate effective antiadhesive, antimicrobial, and antibiofouling properties, including the formation of an oriented monolayer of bacterial flagellin proteins on hydrophobic surfaces [73], a reduction of oral bacteria adhesion on dental brackets by more than 95% due to a reduced surface free energy [74], and the fabrication of antifouling coatings through fluorous media-assisted thermal treatment of stable, hydrophilic protein films [75]. One prominent natural route to prevent the colonization and infection with microbes on cells, tissues, and material surfaces is to block adhesion of bacteria and inhibit attachment to host cells. Microbial cells utilize a variety of extracellular structures such as flagella, pili, fimbriae, and curli fibres, as well as outer membrane proteins to attach to almost every surface [50,76]. During the reversible attachment phase of bacterial biofilm formation, surface pre-conditioning occurs due to soluble organic and inorganic macromolecules that absorb on the underlying materials. The various locomotive appendices, the glycocalyx, and hydrophobic molecules mediating non-specific binding interactions help bacterial attachment even in presence of repulsive forces [77–78]. To address the target protein molecules involved in bacterial adhesion, specific drugs are studied that can disrupt the formation of these molecules. One example are pilicides, derivatives of ring-fused 2-pyridones, which block the correct folding of the pili or fimbriae crucial to bacterial pathogenesis by inhibiting the chaperone–usher polymerization pathway in Gram-negative microorganisms involved in pili protein synthesis [79]. For instance, the inhibitor targets pilus chaperone PapD, thereby reducing the adhesion to cell lines by 90% [80]. These pilicides inhibit curli formation in uropathogenic *E. coli* by preventing the polymerization of protein CsgA and FimC, the chaperone protein of the type-I pili [81]. Once in close contact with mammalian cells, bacteria begin to develop quasi-irreversible attachments by forming stronger bonds with the surface mediated by adhesion proteins or polysaccharides also binding to components of the ECM [50]. The most important adhesion proteins produced by numerous bacteria and virus particles involve surface lectins, known as adhesins and hemagglutinins, that bind to complimentary tissue-specific host cell-surface glycoproteins and glycolipids [82]. Blocking these bacterial lectins by their analogues or suitable carbohydrates has been reported as a possible route for preventing and treating microbial infections [78]. For example, a novel group of adhesins widely found in Gram-negative pathogens has been described, termed multivalent adhesion molecule (MAM7) [83]. The adhesin genes are constitutively expressed, and adhesins are themselves involved in the initial attachment phase of pathogens, by interacting with both the host cell receptor phosphatidic acid and the co-receptor fibronectin. Recombinant production of MAM7 and subsequent coupling on polymer beads has been reported to decrease the surface attachment and infection of pathogens, mimicking the bacterial surface display of the adhesins. Pre-treatment of host cells with MAM7-based inhibitors has shown to inhibit bacterial infection with a range of important clinical pathogens, including *Vibrio parahaemolyticus, Vibrio cholerae, Yersinia pseudotuberculosis*, and enteropathogenic *E. coli* [83–84].

Another protein-based antimicrobial strategy in nature is the use of antimicrobial peptides (AMPs) [85]. These molecules are short-length amphipathic peptide molecules (between 10 and 50 amino acids), usually with cationic charges and hydrophobic residues, and are naturally produced by all multicellular organisms as immune response elements. They are involved in multiple interactions in and outside the cells in response to infections and microbial assault [86]. Mammalian AMPs are generally produced in epithelial tissues of the testis, skin, gastrointestinal tract, and respiratory tract, as well as in leukocyte cells such as monocytes, neutrophils, and others, thus forming a central part of mammalian innate immunity [87]. The multitude of immuno-functional roles of the broad-spectrum antimicrobial peptides includes (1) eliminating bacterial growth through direct antimicrobial activity, (2) inhibiting biofilm-specific signalling pathways, and (3) modulating the innate immune responses resulting in the inhibition of potentially harmful inflammation reactions [85–86,88]. In order to highlight the multifaceted nature of AMPs, the term host defense proteins (HDPs) was coined [89]. HDPs are rich in cationic amino acids (e.g., arginine and lysine), and amino acids with hydrophobic side chains (e.g., tryptophan, phenylalanine, tyrosine, leucine, isoleucine, and valine). This composition renders them amphiphilic, which is crucial to their mode of action and promotes their attachment to the bacterial surface/membrane residues [85]. HDPs have properties of cell-penetrating peptides and can translocate across membranes of prokaryotic, but also eukaryotic, cells [90]. The main mode of action of these peptides is promotion of adhesion through the cationic groups to the anionic bacterial membranes and their lipopolysaccharides (a component of the outer membrane of most Gram-negative bacteria), enabling insertion into the membrane driven by clusters of hydrophobic residues [85,91]. As a consequence, the microbial membranes are permeabilized, associated with membrane perturbation and dissipation of the transmembrane potential, which results in bacterial cell lysis and death [92]. In humans, over 120 HDPs have been identified, and typical examples include lysozyme, defensins, histatins, lactoferricin, kinocidins, ribonuclease, dermcidin, and cathelicidins [93]. In the cathelicidin family of HDPs, named by the ability to inhibit the protease cathepsin L, the amphipathic, helical peptide LL-37 is the only cathelicidin-derived HDP found in humans [87,91]. It was reported that this peptide is able to inhibit *Pseudomonas* biofilm formation at 1/16 of its minimum inhibitory concentration (MIC) for planktonic organisms [94], and, subsequently, it was demonstrated that a distinct subset of HDP peptides can be addressed specifically as antibiofilm peptides, with similar overall amino acid compositions but distinct structure–activity relationships compared to those of antimicrobial peptides [95]. As a result of the multiple functionalities of HDPs, several amphiphilic antimicrobial molecules have recently been developed mimicking both structure and functional aspects of the antimicrobial peptides, such as all-ᴅ amino acid peptides [96], β-peptides [97], and peptide isomers known as peptoids, (oligo N-substituted glycines) [98]. In addition, the transfer of the general molecular structure of AMPs/HDPs has led to peptide-mimicking polymers and surface-engineered polymeric-brush-tethered AMPs. These approaches are promising for establishing biomaterials and medical devices with antifouling and antibacterial properties, but with reduced susceptibility to enzymatic degradation [85]. General advantages of AMPs/HDPs and mimicking polymer systems over antibiotics as a new generation of antimicrobial agents are their functional diversity, broad spectrum of potential practical applications such as tissue repair, the protection of implanted devices, low potential of bacterial resistance due to their multiple bacterial targets, and host/non-host microbe specificity [85–86,99].

#### 1.4 Importance of (protein-based) bioselective materials

One of the biggest threats to human health worldwide is the rapid emergence of multidrug-resistant pathogens, due to the overuse of broad-spectrum antibiotics [100]. The rising rates of antimicrobial and antiviral resistance of infectious bacteria, viruses, and fungi and their related diseases impact all aspects of modern medicine. Recent outbreaks of severe diseases such as Ebola, influenza, and even more so the severe acute respiratory syndrome (SARS) and the pandemic SARS-CoV-2, have tremendously increased the urgency for new concepts and materials that prevent pathogenic microbial infestation and contact-based spreading due to contaminations of surfaces leading to biofilm formation [101]. In contrast, for biomaterials and their biomedical application (such as implants and wound dressings), cell-friendly and adhesive properties are necessary [66]. Ideally, bioselective materials that promote host cell adhesion while simultaneously preventing microbial infestation and biofouling would exhibit a best-of-both-worlds approach for a number of biomedical applications. For instance, it has been shown that the surface modification of glass substrates with the cell-binding motif RGD of fibronectin and collagen significantly enhanced 3T3 fibroblast cell adhesion. RGD further showed no specific binding affinity towards both clinically relevant bacteria *E. coli* or *S. aureus*, and collagen reduced *E. coli*, but enhanced *Staphylococcus aureus* (*S. aureus*) adhesion [102]. Similar results were obtained for chemically coupled RGD and collagen to an antimicrobial polymeric multilayer composed of dextran sulfate and chitosan [103].

One way to achieve bioselective, biocompatible, and multifunctional materials for biomedical applications is the utilization of natural protein materials shown to accomplish the desired properties and derived bio-inspired materials thereof. Protein-based materials are interesting candidates due to their natural origin, biological activity, and structural properties. Among them are silk materials, in particular one made of spider silk proteins [104]. These are well-studied examples characterized by extraordinary properties including excellent biocompatibility, slow biodegradation, low immunogenicity, and non-toxicity, making them ideally suited for tissue engineering and biomedical applications [105–107]. This review focuses on achievements made with silk-based protein materials and highlights the interaction with mammalian cells and microbes as far as the important aspect of bioselective surfaces is concerned.

### 2 Natural silk proteins

#### 2.1 Silk protein classification

The term silk generally refers to extracorporeal, proteinaceous structural materials, mostly in the form of threads [108–109]. Silk proteins evolved around 250 million years ago and are present in a variety of recent arthropod families, most prominently in silkworms and spiders, but also in bees, wasps, and ants [110]. Silks fulfill numerous functions, including prey capture, dispersal, reproduction, adhesion, and building cocoons, nests, egg sacs, and stalks [6,110]. Silk proteins are biochemically diverse but do share common elements. They usually consist of large proteins that contain long motifs of highly repetitive amino acid sequences rich in alanine, serine, and/or glycine [111]. Moreover, the proteins are stored as highly concentrated silk dope solutions in specialized glands and solidify in a spinning process often associated with shear forces accompanied by their folding into a single dominant secondary structure [112]. Due to both convergent evolution (i.e., silks have been “invented” independently several times), and adaptation to specific functions, the amino acid sequence and protein structure varies considerably, resulting in a versatile class of proteins [112]. Silks are known as semicrystalline materials since they consist of ordered, crystalline structures embedded in an amorphous matrix. The regular secondary structure within one type of silk allows for the condensed packing of protein, as well as the formation of hydrogen bonds, leading to tightly connected intra- and inter-protein chains [111]. While the crystalline regions exhibit a high hydrogen bond density accounting for the strength of a silk fibre, the unordered amorphous regions with less hydrogen bond density induce flexibility [109].

Besides considerable variations in arthropods, the silk of silkworms and related moths, and that of orb-weaving spiders share some features. Their silk proteins are often of high molecular weight, and whilst the termini are hydrophilic, the repetitive cores are composed of alternating large hydrophobic amino acid sequence blocks interspersed with short hydrophilic parts. Interestingly, in both insect (fibroin) and spider (spidroin) silk fibres the core is composed of fibrils that are oriented along the fibre axis and contain nanometre-sized β-sheet crystallites [113–114]. Silkworm silk contains mainly three different proteins, namely a heavy chain fibroin, a light chain fibroin linked via disulfide bonds to the former one, and a P25 glycoprotein associated via noncovalent hydrophobic interactions as a putative stabilizer of the fibre complex integrity [115]. Recently, it has been reported that the accessory Filippi’s glands of silkworms appear to regulate posttranslational modifications of fibroin heavy chain molecules, necessary for a tight cocoon architecture [116]. Silk fibroins make up to 75–83 wt % of raw silkworm silk, while sericins, hydrophilic proteins forming a connective coating, constitute 17–25% of the weight of the fibre. Fibroins contain very few cysteine residues, whereas glycine, alanine, serine, and tyrosine make up more than 90 mol %. The fibroins largely contain blocks of (GAGAGS)*_n_*, which are responsible for the anisotropic β-sheet-rich nanocrystals [108]. Sericins are based on glue-like serine-rich glycoproteins with rubber-elastic properties [110]. In addition, a further small protein group named seroins was discovered in lepidopteran silk and is known to possess AMP-like antimicrobial activities. It is produced in the silk gland, but also to a lesser extent in the midgut and fat body [117]. As a consequence of its immunogenic and allergic properties, the sericin coating of silkworm silk fibres has to be removed before further processing or use in medical applications in a process known as degumming [108,112].

Spider silks display a much higher degree of diversity, with up to seven different silk types found in the more highly derived group of orb web spiders [108]. This enables spiders to produce mechanically stable web constructions as well as dragline fibres and, at the same time, to wrap their eggs and utilize sticky glue silk for prey capture. The different silks are named after the spidroin-producing spinning glands, such as major and minor ampulla, flagelliform, aggregate and piriform glands, which produce the scaffold thread, an auxiliary spiral, the catching spiral and its glue, and the net anchors. Aciniform and cylindriform silk, in contrast, are used for the egg case [118]. Spider silk consists of 99% spidroins plus some additional lipids, glycoproteins, and ions such as phosphate. The core domain of spidroins consists of short motifs with 10–50 amino acids, which are present in larger modular units, often copied up to several hundred times, giving spidroins a high molecular weight of several hundred kilodaltons [109]. The core domains are flanked by two globular terminal domains, both highly conserved and very similar in many spider species [119]. These globular domains fulfill important control functions concerning protein solubility in the gland reservoir and in the assembly of spidroins into silk threads. The various spidroins of spiders differ in their amino acid sequences to yield the specific properties and functions of the silks. However, some conserved motifs like polyalanine (A*_n_*, poly-A), glycine–alanine (GA), glycine–glycine–X (GGX) and glycine–proline–glycine–(X)*_n_* (GPG(X)*_n_*, where X is typically tyrosine, leucine, or glutamine) are abundant in the repetitive core regions in various spidroins [120]. Similar to silkworm silk, poly-A and GA sequences in the spider dragline thread form nanocrystalline hydrophobic β-sheets aligned along the fibre axis, which are embedded in a matrix of more hydrophilic GGX and GPG(X)*_n_* regions responsible for helical and spring-like secondary structures with fewer hydrogen bonds and higher elasticity [114]. The best-studied silk type is the major ampullate silk (MA) used by spiders for web radii and their dragline. It consists of several proteins known as major ampullate spidroins (MaSps). The most prominent spidroins are MaSp1 and MaSp2, differing mainly in their proline content [114], while in some spiders also short MaSp1 variants, and MaSp3 and MaSp4 proteins have been identified [121–122].

#### 2.2 Silk-based materials and their properties

Materials based on arthropod silk proteins attracted increasing attention among material scientists in the last decades. The prominent cocoon silk of several silk moths, produced for the wrapping of cocoons during metamorphosis from larvae to adult moths, has been used by humans for millennia [108]. The application of silkworm silk covers a wide range from traditional textiles to technical fabrics and cosmetic articles including skin care, shampoos, and lotions. Traditional utilization of silk fibres as sutures for wounds are known for centuries due to their strength, biocompatibility, and low immunogenicity [104], and silkworm silk surgical threads are commercially available [108]. Moreover, silk-based polymeric materials have also been reported to be suitable as tissue scaffolds due to their biocompatibility and their highly tunable morphologies and mechanical properties. Exemplary, silkworm silk has been studied as a matrix material for tissue-engineered anterior cruciate ligaments [123], and silk fibroin/nanohydroxyapatite composite hydrogels have been studied regarding bone tissue engineering [124].

Spider silk has also been widely used as a multifunctional material for fishing nets, wound coverings, and sutures for surgery for centuries in Australasia and Greece [108]. Since then, it came in focus of researchers worldwide. Natural spider silk has the potential to be used for tissue engineering and biomedical applications. For instance, NIH 3T3 cells showed increased adhesion and proliferation on fabricated, sterilized wovens made of native, unmodified spider dragline silk fibres from *Nephila clavipes* over five days [125]. Furthermore, Wendt et al. showed that naturally occurring spider dragline silk fibres from *Nephila* species could be processed into wovens for culturing first fibroblasts for two weeks before seeding keratinocytes on top to develop a bilayered skin model consisting of an artificial dermis and epidermis [126].

Strikingly, silk materials from silkworms and spiders share a set of highly interesting properties, including mechanical and chemical stability, biocompatibility for mammalian cells and tissues, persistence against microbial degradation and, moreover, potential microbe-repellent and antimicrobial features [50]. Several studies reported antibacterial properties, reduced biofilm formation, and bacterial repellency of both natural silkworm [127–129] and spider silk threads and cocoons [130–136]. However, the status of the intrinsic properties of silk materials in the context of microbial defense is a matter of debate in the scientific community [137–138]. While some of the inconsistencies seem to be related to methodological aspects of the reports in doubt, a misunderstanding of the distinction between the three mechanisms of biofilm inhibition, namely microbial repellency, microbiocidal activity, and contact killing, may foster the discussion on the anti-fouling properties of silk protein materials [50]. Concerning spider silk materials, it has been reported that they seem to prevent bacterial attachment to the surface and biofilm formation instead of being antimicrobial [136].

### 3 Engineered silk protein-based materials

#### 3.1 Recombinant spider silk technology

The unique properties of spider silk make it a promising candidate for biomedical and tissue engineering applications [105–107]. However, the amounts that could be harvested from spiders are low, and the farming of most spiders is not possible due to their territorial behaviour. Therefore, different recombinant production systems have been developed over time to generate spider silk proteins in appropriate amounts and quality [139–140]. The Johansson research group used the amino acid sequence of the spider *Euprosthenops australis* as inspiration and developed the recombinant spider silk protein 4RepCT comprising four repetitions of a sequence with a polyalanine-rich and a glycine-rich block, as well as a non-repetitive C-terminal domain [141–142]. Another recombinant system is based on the dragline silk of the European garden spider *Araneus diadematus*: Two native spidroins of this silk, namely *Araneus diadematus* fibroin (ADF) 3 and 4, have been used as blueprints for generating the engineered variants eADF3(AQ12) and eADF4(C16). Both proteins contain polyalanine-rich sequences, but eADF4(AQ12) contains two sequence modules (A and Q), while eADF4(C16) comprises only one module (C), repeated either twelve or 16 times, respectively, to generate artificial spider silk proteins [143]. Due to a glutamic acid residue in the C-module, eADF4(C16) is negatively charged [143]. Replacing this amino acid yielded a positively charged eADF4(κ16) and an uncharged eADF4(Ω16) variant, where the glutamic acid residue is exchanged with a lysine or glutamine residue, respectively [144–146]. The dragline silk of *Nephila clavipes* inspired the recombinant spider silk proteins 6mer and 15mer, in which the consensus sequence is repeated six or 15 times, respectively [147]. These recombinant spider silk proteins have been developed using genetic engineering to generate the appropriate gene sequence of interest. In all these cases, recombinant protein production was achieved using bacteria (*E. coli*) [141–143,147]. The usage of genetic engineering further allows for diverse modifications on the genetic level, including site-specific amino acid changes leading to differently charged proteins [144–146] and the addition of short amino acid sequences [114,148–154] or whole domains [155–157] and even proteins [158–160].

#### 3.2 Cell adhesive silk material surfaces

Since the engineered ADF4 sequences lack cell binding sequences, biological interaction and cell attachment is quite limited to recombinant spider silk materials made thereof [161]. Cell culture studies on film surfaces using Balb 3T3 fibroblasts [148], neonatal rat cardiomyocytes [162], human induced pluripotent stem cell (hiPSC) derived cardiomyocytes [150], keratinocytes, myoblasts, or neuronal cells [163] revealed that negatively eADF4(C16) and uncharged eADF4(Ω16) surfaces do not allow good and sufficient cell attachment necessary for tissue engineering applications. However, several independent studies showed that the materials made of the positively charged eADF4(κ16) variant enabled partial cell adhesion due to the positive surface charge derived from lysine residues in the primary amino acid sequence [150,162–163]. Since cells expose many negatively charged molecules on their cellular surface, charge modification is often used to guide cellular interaction [164–166]. In the example of the recombinant 4RepCT spider silk, several charged amino acid residues in the primary protein sequence create a material surface that allows for the growth of primary fibroblasts without further modification [167].

**Figure 3:**
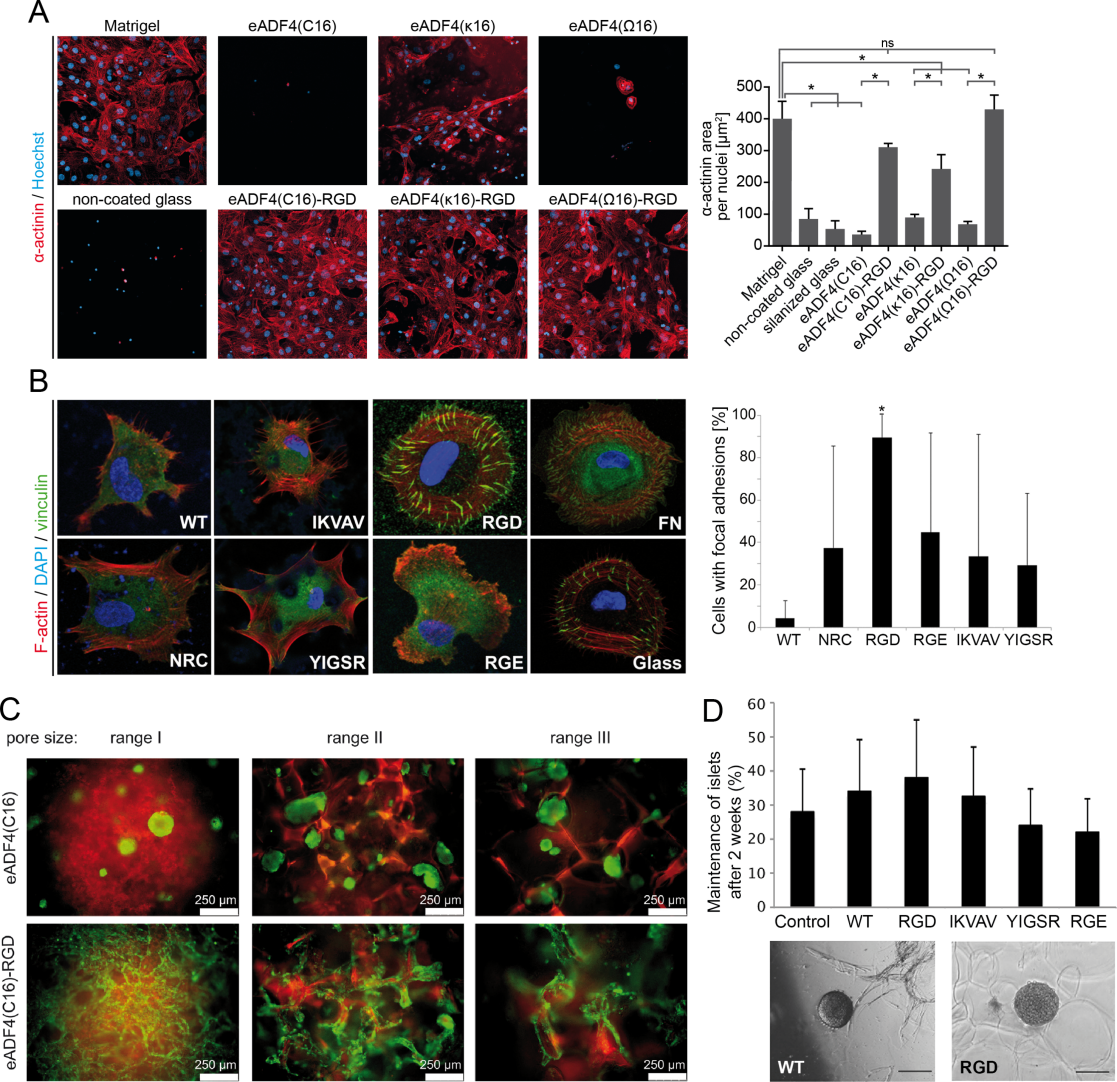
Bioselective, cell adhesive spider silk surfaces. (A) hiPSC-derived cardiomyocytes adhere and spread on spider silk films made of eADF4(C16)-RGD, eADF4(κ16), eADF4(κ16)-RGD, and eADF4(Ω16)-RGD and on control films made of Matrigel. Figure 3A was reprinted from reference [150], Materials Today Bio, vol. 11, by T. U. Esser; V. T. Trossmann; S. Lentz; F. B. Engel; T. Scheibel, “Designing of spider silk proteins for human induced pluripotent stem cell-based cardiac tissue engineering”, article no. 100114, Copyright (2021) The Authors, with permission from Elsevier. This content is not subject to CC BY 4.0. (B) Human dermal fibroblasts attachment and spreading behaviour on unmodified (WT) and peptide (NRC, RGD, RGE, IKVAV, and YIGSR)-functionalized 4RepCT spider silk films compared to control surfaces (glass and fibronectin (FN)) after 3 h. Figure 3B was reprinted with slight adaptation from reference [152], Biomaterials, vol. 34, issue 33, by M. Widhe; U. Johansson; C.-O. Hillerdahl; M. Hedhammar, “Recombinant spider silk with cell binding motifs for specific adherence of cells”, pages 8223-8234, Copyright (2013), with permission from Elsevier. This content is not subject to CC BY 4.0. (C) Balb 3T3 fibroblasts showed enhanced adhesion, spreading, and elongation on foams made of eADF4(C16)-RGD (lower panel) compared to unmodified eADF4(C16) ones (upper panel) independent of the pore size. Figure 3C was reprinted with permission from [169], Copyright 2016 American Chemical Society. This content is not subject to CC BY 4.0. (D) Human pancreatic islets could adhere, survive, and maintain their function on functionalized foams made of unmodified (WT) and peptide (RGD, RGE, IKVAV, and YIGSR)-functionalized 4RepCT foams. Figure 3D was adapted from reference [154] (© 2015 U. Johansson et al., published by PLOS, distributed under the terms of the Creative Commons Attribution 4.0 International License, https://creativecommons.org/licenses/by/4.0/).

As mentioned above, bioselective cell adhesion could be generated by attaching or introducing binding sites, for example, peptide sequences such as RGD, for specific interaction with cellular receptors [41]. In case of recombinant eADF4-based silk, the RGD peptide was genetically (C-terminal) and chemically (N-terminal) coupled to eADF4(C16) to selectively allow for cell attachment on materials made thereof. Fusing the integrin non-binding peptide RGE, showing a residue substitution of aspartate with glutamate thus rendering an identical charge distribution, was applied as a control. Cell culture studies using Balb 3T3 fibroblasts revealed that the RGD peptide enhanced primary cell attachment with increased spread morphology and proliferation on films independently of chemical or genetic modification. The unmodified eADF4(C16) and eADF4(C16)-RGE control surfaces showed less or no cell interaction [148]. In addition, eADF4(C16)-RGD films allowed for adhesion and spreading of neonatal rat cardiomyocytes and hiPSC-derived cardiomyocytes, and further supported their cellular functions, such as beating and contraction [150,168]. The C-terminal genetic modification of the uncharged eADF4(Ω16) and positively charged eADF4(κ16) variant with RGD also enabled the attachment of hiPSC-derived cardiomyocytes and the development of their cell-specific functions on flat films (Figure 3A) [150]. In another study, eADF4(C16) was modified with biomineralization and collagen-binding peptides, yielding an increased osteoblast attachment on these functionalized variants [149].

Genetic modification of 4RepCT led to recombinant spider silk proteins containing linear RGD, RGE, IKVAV, and YIGSR sequences at their N-terminal end. Cell culture analysis using primary cells (fibroblasts, keratinocytes, endothelial, and Schwann cells) revealed that these peptide tags had a clear positive impact on early cell attachment and spreading (Figure 3B). Especially, adhesion and spreading of Schwann cells were influenced in case of the IKVAV modification. Nevertheless, after a longer incubation time (24 h) all modified surfaces supported cell growth [152]. Another study compared the influence of different N-terminally fused RGD-containing peptides on cell behaviour (keratinocytes, endothelial, and human mesenchymal stem cells). It could be shown that all RGD peptides enhanced the adhesiveness of these spider silk surfaces, but the disulfide-bridged, fibronectin-derived integrin-binding RGD peptide showed the highest impact on cell adhesion [151]. RGD-modified FN-4Rep-CT silk could also be used to generate self-assembled membranes, allowing for the adhesion of endothelial cells on the one side and smooth muscle cells on the other side, as well as the diffusion of relevant molecules, making this material promising for vascular tissue engineering [170].

In addition to flat films or coatings, recombinant spider silk proteins based on eADF4 can be processed into 3D scaffolds, such as foams or hydrogels due to their self-assembly properties [169,171–174]. Balb 3T3 fibroblasts adhered to porous foams made of eADF4(C16)-RGD and showed spreading and cell elongation along the foam walls, while cellular aggregates were formed in and on unmodified eADF4(C16) foams, because the cells preferred cell-cell-contacts over cell-material-interactions (Figure 3C) [169]. Additionally, it could be shown that osteoblasts, fibroblasts, keratinocytes, and myoblasts adhere to eADF4(C16)-RGD hydrogels, while they could not attach to unmodified eADF4(C16) hydrogels [174]. A previously published in vivo study of an arteriovenous loop model in rats showed that RGD-functionalized spider silk hydrogels significantly enhanced angiogenesis by forming new blood vessels compared to unmodified eADF4(C16) hydrogels [175].

4RepCT could also be processed in 3D foams for cell culture analysis [154,167,176]. Foams of the genetically modified 4RepCT variants carrying RGD, RGE, IKVAV, and YIGSR sequences were analysed in presence of pancreatic cells for insulin production. While the adhesion and formation of islets was clearly enhanced on RGD-modified foams, a high insulin production was measured for RGD- and YIGSR-modified foams after two days. Although all spider silk scaffolds could be used for culturing pancreatic cells with long-term maintenance of function, the bioactive RGD peptide modification showed the most promising results for in vivo applications (Figure 3D) [154]. One other study analysed the impact of the location of RGD sequences inside the 4RepCT protein on cell behaviour. Therefore, different RGD-containing peptides were genetically introduced at the N-terminus or between repetitive units of the 4RepCT silk protein. The analysis using pancreatic islet cells revealed that peptides inside the protein sequence enhanced cluster formation and total number of these islets, but the viability and functionality of the clusters was enhanced on all RGD-modified substrates [176]. The multitude of studies using functionalized recombinant spider silk in tissue engineering confirmed the high impact and suitability of bioadhesive spider silk protein-based materials for applications in that field.

#### 3.3 Antimicrobial silk-based surfaces

**Figure 4:**
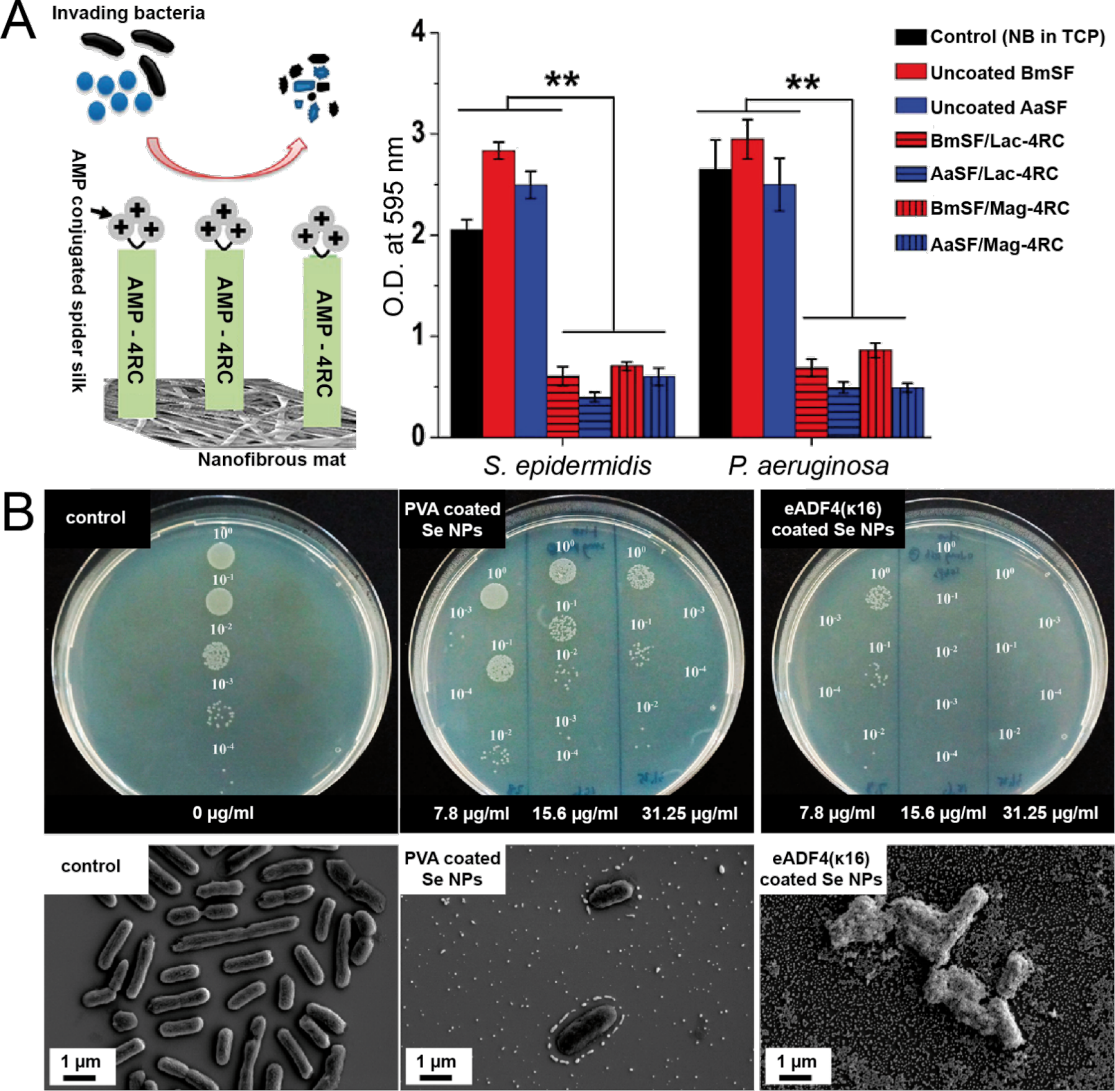
Antimicrobial spider silk materials. (A) Nanofibrous silk fibroin mats (BmSF and AaSF) are coated with recombinant 4RepCT spider silk proteins functionalized with magainin I (Mag-4RC) or lactoferricin (Lac-4RC) cationic antimicrobial peptides. Crystal violet staining (O.D. at 595 nm) of *S. epidermidis* and *P. aeruginosa* bacteria reveals significantly lower biofilm formation on spider silk-coated silk fibroin mats compared to uncoated ones. Figure 4A was adapted with permission from [183], Copyright 2018 American Chemical Society. This content is not subject to CC BY 4.0. (B) Images of agar plates of a colony-forming unit assay and representative scanning electron images of *E. coli* bacteria without and after a treatment using polyvinyl alcohol (PVA) and eADF4(κ16) spider silk-coated selenium (Se) nanoparticles (NPs) in comparison to a particle-free control. Concentrations as indicated. 10^0^ represents the original solution of bacteria without or with appropriate particles, while 10^−1^, 10^−2^, 10^−3^, and 10^−4^ indicate, respectively, 10-, 100-, 1000- and 10000-fold dilutions of the original suspension. Figure 4B was adapted from reference [184] (“Enhanced Antibacterial Activity of Se Nanoparticles Upon Coating with Recombinant Spider Silk Protein eADF4(κ16)“, © 2020 Huang et al., published and licensed by Dove Medical Press Ltd., distributed under the terms of the Creative Commons Attribution-NonCommercial 3.0 Unported License, http://creativecommons.org/licenses/by-nc/3.0/). This content is not subject to CC BY 4.0.

As already pointed out in section 2.2, no uniform hypothesis has been formulated so far for spider silk surfaces concerning their microbial repellence and persistence against microbial degradation. For example, one study reported increased bacterial growth on spider silk webs of *Linothele fallax* and *Linothele megatheloides* and discussed that spider silk proteins may serve as nutrients for various microbes [177], whereas other studies provided clear evidence that spider silks of the species *Tegenaria domestica, Nephila pilipes, Hippasa holmerae*, and *Cyrtophora moluccensis* are microbe-repellent [131,136]. More species have been studied in other reports, and as one result thereof it is clear that microbial repellence is not a general trait in spider silks [178]. One way to characterize this issue is through the analysis of genetically engineered silk proteins, allowing for a precise control of the design and modifications of the underlying amino acid sequence motifs and the structure–property relationships of tailored silk materials. Thus, intrinsic antimicrobial properties of materials based on recombinant silk proteins can also be analysed in greater detail. Modifications with specific functions, for example, AMPs against biofouling, enzymes, and nanoparticles, open further roads towards antimicrobial or repellent protein-based materials. As an example, Harris et al. studied coatings made of recombinant spider silk proteins based on the dragline silk amino acid sequence of *Nephila clavipes* MaSp1 and MaSp2 on a variety of substrates [179]. The authors reported an intrinsic ability of the recombinant spider silk proteins to prevent biofouling due to bacterial manifestation. In addition, functionalization of the silk proteins with antimicrobial biologicals displayed increased inhibition of surface fouling [179]. Fusion proteins combining recombinant MaSp1 spider silk proteins from *Nephila clavipes* with the AMPs human neutrophil defensin 2 and 4 (HNP-2, HNP-4) and hepcidin, showed antimicrobial activity against Gram-negative *E. coli* and Gram-positive *Staphylococcus aureus* [180]. A more recent study reported on the fusion protein 2Rep-HNP1 consisting of two repeats of the *Euprosthenops australis* MaSp1 repetitive core region and the AMP human neutrophil defensin 1 active peptide (HNP-1) to create antimicrobial silk as a promising new suture material [181]. The tagged 2Rep-HNP1 retained a broad-spectrum antimicrobial activity above 90% against the same bacterial pathogens mentioned above [181]. Another approach aimed to take advantage of the combined usage of natural silk fibroin as a bulk scaffold and a coating with recombinant spider silk proteins functionalized with specific modifications [182]. The recombinant spider silk protein 4RepCT was fused with two AMP motifs with antimicrobial activity, namely magainin I (Mag-4RC) and lactoferricin (Lac-4RC), successfully inhibiting biofilm development of the skin-infecting bacteria *Pseudomonas aeruginosa* (*P. aeruginosa*) and *Staphylococcus epidermidis* (Figure 4A) [183].

Introducing antimicrobial properties on surfaces and coatings using antibacterial agents in silks is an advantageous route to reduce or inhibit microbial infestation. The concentration of the antibacterial agent could be locally increased without exceeding toxicity limits as opposed to conventional antibiotic delivery methods in solution. One approach to make materials antimicrobial is to implement metallic nanoparticles in the bulk material. Silver ions in solution have shown antibacterial properties and are widely used in medicine [185]. The release of silver ions from silver nanoparticles (AgNPs) resulted in enhanced chemical, physical, and biological properties [186–187]. In addition to bactericidal properties, physical damage of bacterial membranes by AgNPs was also observed, causing cell leakage and, eventually, cell death [187–188]. A variety of studies focused on the modification of silkworm silk, including plateless silver coating techniques [189] and various coupling mechanisms of silver or gold nanoparticles [190–192]. In addition, the modification of regenerated sericin using crosslinking or surface-linking to titanium dioxide nanoparticles displayed strong activity against different microbes, resulting in loss of cell integrity and subsequent cell death [193–195]. AgNPs produced with sericin as a reducing agent in situ effectively inhibited the growth of *E. coli*, *S. aureus*, and *P. aeruginosa* in radial diffusion assays [194–195].

The genetic introduction of silver binding sites into recombinant spider silk proteins from *Nephila clavipes* MaSp1 dragline silk has been reported, for example, by Currie and co-workers [196]. The study confirmed the effective chimeric coupling of silver ions from a solution, resulting in potent inhibitory effects on both Gram-positive and Gram-negative bacteria [196]. Apart from silver, carbon in the shape of nanodiamonds incorporated into electrospun silk nanofibres showed antibacterial behaviour [197]. As another example, the antibacterial properties of selenium nanoparticles could further be enhanced by coating them with positively charged recombinant eADF4(κ16) silk proteins (Figure 4B) [184]. In addition, cytotoxicity testing showed that the coated selenium particles are safe to use at certain concentrations in biological applications since they showed acceptable cytotoxicity on Balb 3T3 mouse fibroblasts and HaCaT human skin keratinocytes [184]. Composite films and hydrogels made of recombinant eADF4(C16) spider silk and mesoporous silica nanoparticles (MSN) loaded with the aminoglycoside antibiotics gentamycin, neomycin, and kanamycin as well as the antimycotic amphotericin B, exhibited excellent antimicrobial properties [198]. Surface antimicrobial activities against *E. coli* and *Pichia pastoris* with sustained release of antibiotics could be monitored over the course of 15 days, even at their MIC [198]. Furthermore, the inclusion of antibiotics/antimycotics did not impair the cytocompatibility of the composite materials and promoted fibroblast cell adhesion and proliferation [198].

By incorporating the non-natural methionine analogue ʟ-azidohomoalanine (L-Aha) into the recombinant 4RepCT spider silk protein, site-specific chemical incorporation of two fluorophores and an antibiotic was conducted using copper-catalysed azide–alkyne cycloaddition (a type of click chemistry) [199]. The assembled silk fibres decorated with the broad-spectrum antibiotic levofloxacin showed significant antibiotic activity over at least five days [199]. In another approach, the same recombinant spider silk was successfully functionalized with bacteriolytic or anti-biofouling enzymes for bioactive surface coatings [200]. The group developed recombinant 4RepCT spider silk proteins functionalized with the peptidoglycan degrading endolysin SAL-1 from the staphylococcal bacteriophage SAP-1 and the biofilm-matrix-degrading enzyme dispersin B from *Aggregatibacter actinomycetemcomitans* [200].

#### 3.4 Antiadhesive properties

Besides antimicrobial strategies with a limited number of microbe-killing agents, antiadhesive, anti-fouling, and/or microbe-repellent surfaces are a long-term antimicrobial approach. To this end, it was exemplarily demonstrated that implants and catheters coated with the recombinant spider silk protein eADF4(C16) show significantly reduced adhesion and proliferation of mammalian cells compared to untreated ones [163,201–202]. When transplanted into rats in vivo, eADF4(C16)-coated medical grade silicone implants showed a significant reduction in periprosthetic fibrous capsule formation. Furthermore, the recombinant spider silk protein coatings acted as a bioshield, improved the biocompatibility and significantly decreased the foreign body response to the implant [201–202]. Additionally, it was shown that coatings made of eADF4(C16) silk protein on silicone surfaces could also enhance microbe-repellent properties against four opportunistic infection-related strains, leading to reduced microbial adherence up to 99.7% in comparison to uncoated silicone surfaces [203]. Zhang et al. reported a different repellent effect, due to triboelectric charging of a film made from recombinant spider silk proteins based on a MaSp1 sequence [204]. A potential difference between the positively charged surface and the bacteria was generated by their self-powered triboelectric nanogenerator, which builds up electrical charges of up to 135 V. An extracellular transmission of electrons impairs the bacterial morphology and leads to death of the bacteria by inducing a burst of reactive oxygen species inside the bacterial cytoplasm [204].

Spider silk proteins have also been analysed as far as thrombosis and blood coagulation is concerned. Weiss et al. analysed the effect of surface net charge of several recombinant spider silk variants on the formation of the biomolecular corona in contact with whole blood [205]. The study could show that negatively charged or almost neutral spider silk material surfaces prevented blood clotting, while the positively charged ones interacted predominantly with fibrinogen-based proteins, correlating with increased blood clotting [205]. In another study, silk proteins were functionalized with heparin to prevent thrombosis, reporting an intrinsic ability of the recombinant spider silk proteins to prevent blood clotting [179]. Recently, a heparin-binding and antimicrobial mimicking peptide based on the consensus heparin binding amino acid motif (HBM) was genetically linked to a recombinant MaSp2-based spider silk protein [206]. The fusion protein displayed heparin binding and anticoagulant properties, as well as an intrinsic property to inhibit bacterial growth due to the antimicrobial properties [206].

**Figure 5:**
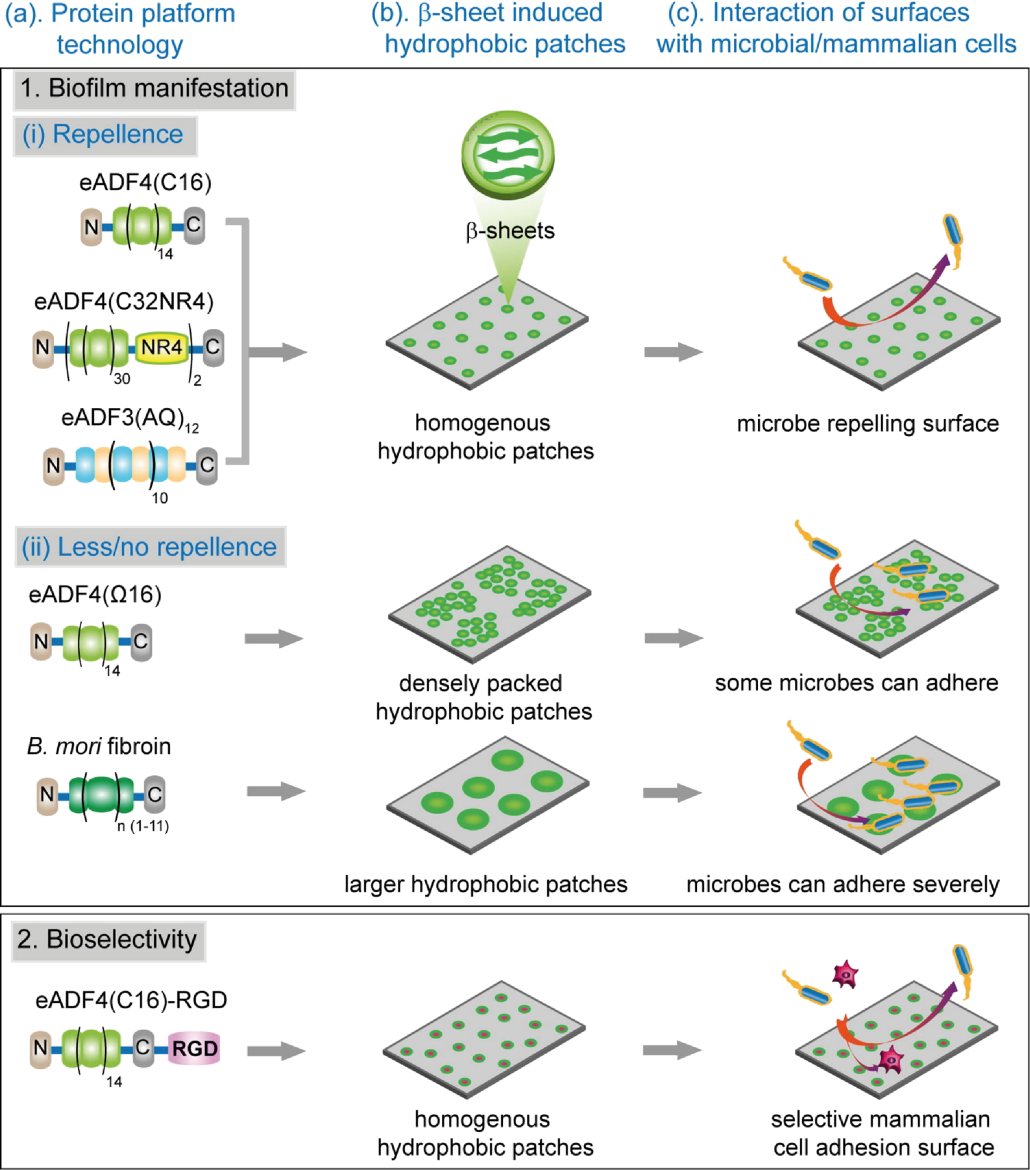
Engineered spider silk proteins for controlled microbial repellence and bioselective cell attachment. Compared to natural silk fibroin from *Bombyx mori*, the recombinant spider silk protein eADF4(C16) shows microbe-repellent behaviour and could be genetically modified (eADF4(C16)-RGD) to selectively allow for the attachment of mammalian cells to generate a multifunctional surface for biomedical applications. Figure 5 was reproduced from reference [146], (© 2020 Kumari et al., published by Elsevier B. V., distributed under the terms of the Creative Commons Attribution-Non-Commercial-NoDerivatives 4.0 International Licence, https://creativecommons.org/licenses/by-nc-nd/4.0/). This content is not subject to CC BY 4.0.

Finally, a systematic study of different variants of engineered *Araneus diadematus* spidroins ADF3 and ADF4 aimed to demonstrate that the intrinsic microbe-repellence of eADF4(C16) spider silk can be altered by a single amino acid exchange in the repetitive unit. The change of negatively charged glutamate in eADF4(C16) to uncharged glutamine in eADF4(Ω16) resulted in nano-structural changes in films and hydrogels, which in turn resulted in the attachment of some microbes [145–146]. It was concluded that size and distribution of crystalline patches within the silk surface structure are crucial to create anti-fouling, microbe-repellent surfaces, in line with the argumentation that antifouling effects of spider silk surfaces are due to nanostructural features of silk [145–146,207]. Simultaneously, bioselectivity of the silk protein material was achieved by introducing the cell adhesion motif RGD to eADF4(C16), resulting in selective mammalian cell adhesion [145–146]. Figure 5 shows a summary of this recombinant spider silk protein approach for bioselective, microbe-repellent surface effects in comparison to silk fibroin.

## Conclusion

Combining microbial repellence with bioselective cell binding is a crucial step towards multifunctional and specific biomaterial applications. A controllable protein-based platform could provide a long-term sustainable and environmentally friendly (in patients and globally) material for this approach. Thus, there is great interest in developing advanced green materials that inhibit microbial adhesion to surfaces while simultaneously promoting selective mammalian cell growth. Arthropod silk materials, and, in particular, spider silk and their recombinant protein mimics, have been demonstrated to be ideally suited candidates to this end. Established recombinant spidroins provide defined protein composition and structure–function relationships, as well as the possibility to incorporate modifications for tailored functionality. Furthermore, such spidroins could be processed into several material forms and are inherently biocompatible and non-toxic. Thus, engineered spider silks provide a huge potential for future biomaterial applications due to the large variety of possible modifications, for instance, the integration of various cell binding motifs or AMPs. These important properties demonstrate the great ability of spider silk-based materials for the usage as bioselective, microbe-resistant coatings for technical as well as biomedical applications and tissue engineering.

## References

[1] Fazleabas A T, Kim J J (2003). Science.

[2] Keckes J, Burgert I, Frühmann K, Müller M, Kölln K, Hamilton M, Burghammer M, Roth S V, Stanzl-Tschegg S, Fratzl P (2003). Nat Mater.

[3] Melzer B, Steinbrecher T, Seidel R, Kraft O, Schwaiger R, Speck T (2010). J R Soc, Interface.

[4] Hofman A H, van Hees I A, Yang J, Kamperman M (2018). Adv Mater (Weinheim, Ger).

[5] Gorb S N (2008). Philos Trans R Soc, A.

[6] Grunwald I, Rischka K, Kast S M, Scheibel T, Bargel H (2009). Philos Trans R Soc, A.

[7] Smith A M (2016). Biological Adhesion.

[8] Rozario T, DeSimone D W (2010). Dev Biol (Amsterdam, Neth).

[9] Petruzzelli L, Takami M, Humes H D (1999). Am J Med.

[10] Harjunpää H, Llort Asens M, Guenther C, Fagerholm S C (2019). Front Immunol.

[11] Sperandio M (2006). FEBS J.

[12] Samanta D, Almo S C (2015). Cell Mol Life Sci.

[13] Aplin A, Howe A, Alahari S, Juliano R (1998). Pharmacol Rev.

[14] Patel S D, Chen C P, Bahna F, Honig B, Shapiro L (2003). Curr Opin Struct Biol.

[15] Yap A S, Brieher W M, Gumbiner B M (1997). Annu Rev Cell Dev Biol.

[16] Wai Wong C, Dye D E, Coombe D R (2012). Int J Cell Biol.

[17] Cayrol R, Wosik K, Berard J L, Dodelet-Devillers A, Ifergan I, Kebir H, Haqqani A S, Kreymborg K, Krug S, Moumdjian R (2008). Nat Immunol.

[18] Kappelmayer J, Nagy B (2017). BioMed Res Int.

[19] Zhao J, Santino F, Giacomini D, Gentilucci L (2020). Biomedicines.

[20] Hynes R O (2009). Science.

[21] Berrier A L, Yamada K M (2007). J Cell Physiol.

[22] Legate K R, Wickström S A, Fässler R (2009). Genes Dev.

[23] Han W M, Jang Y C, García A J, Wagner W R, Sakiyama-Elbert S E, Zhang G (2020). The Extracellular Matrix and Cell–Biomaterial Interactions. Biomaterials Science.

[24] Pankov R, Yamada K M (2002). J Cell Sci.

[25] De Marco R, Greco A, Calonghi N, Dattoli S D, Baiula M, Spampinato S, Picchetti P, De Cola L, Anselmi M, Cipriani F (2018). Pept Sci.

[26] Zhang J, Jiang X, Wen X, Xu Q, Zeng H, Zhao Y, Liu M, Wang Z, Hu X, Wang Y (2019). JPhys Mater.

[27] Mano J F (2008). Adv Eng Mater.

[28] Gagner J E, Kim W, Chaikof E L (2014). Acta Biomater.

[29] Choi S M, Chaudhry P, Zo S M, Han S S, Chun H J, Park C H, Kwon I K (2018). Advances in Protein-Based Materials: From Origin to Novel Biomaterials. Cutting-Edge Enabling Technologies for Regenerative Medicine.

[30] Annabi N, Mithieux S M, Camci-Unal G, Dokmeci M R, Weiss A S, Khademhosseini A (2013). Biochem Eng J.

[31] Shin H, Jo S, Mikos A G (2003). Biomaterials.

[32] Spiller S, Clauder F, Bellmann-Sickert K, Beck-Sickinger A G (2021). Biol Chem.

[33] Hosoyama K, Lazurko C, Muñoz M, McTiernan C D, Alarcon E I (2019). Front Bioeng Biotechnol.

[34] Huettner N, Dargaville T R, Forget A (2018). Trends Biotechnol.

[35] Yamada K M (1991). J Biol Chem.

[36] Pountos I, Panteli M, Lampropoulos A, Jones E, Calori G M, Giannoudis P V (2016). BMC Med.

[37] Ruoslahti E, Pierschbacher M D (1986). Cell.

[38] Pierschbacher M D, Hayman E G, Ruoslahti E (1985). J Cell Biochem.

[39] Hersel U, Dahmen C, Kessler H (2003). Biomaterials.

[40] Pierschbacher M D, Ruoslahti E (1984). Nature.

[41] Ruoslahti E (1996). Annu Rev Cell Dev Biol.

[42] Bellis S L (2011). Biomaterials.

[43] Pfaff M (1997). Recognition Sites of RGD-Dependent Integrins. Integrin-Ligand Interaction.

[44] Mertgen A-S, Trossmann V T, Guex A G, Maniura-Weber K, Scheibel T, Rottmar M (2020). ACS Appl Mater Interfaces.

[45] Rechenmacher F, Neubauer S, Mas-Moruno C, Dorfner P M, Polleux J, Guasch J, Conings B, Boyen H-G, Bochen A, Sobahi T R (2013). Chem – Eur J.

[46] Grant D S, Tashiro K-I, Segui-Real B, Yamada Y, Martin G R, Kleinman H K (1989). Cell.

[47] Graf J, Iwamoto Y, Sasaki M, Martin G R, Kleinman H K, Robey F A, Yamada Y (1987). Cell.

[48] Graf J, Ogle R C, Robey F A, Sasaki M, Martin G R, Yamada Y, Kleinman H K (1987). Biochemistry.

[49] Sullivan T, O’Callaghan I (2020). Biomimetics.

[50] Sonnleitner D, Sommer C, Scheibel T, Lang G (2021). Mater Sci Eng, C.

[51] Ivanova E P, Hasan J, Webb H K, Truong V K, Watson G S, Watson J A, Baulin V A, Pogodin S, Wang J Y, Tobin M J (2012). Small.

[52] May R M, Magin C M, Mann E E, Drinker M C, Fraser J C, Siedlecki C A, Brennan A B, Reddy S T (2015). Clin Transl Med.

[53] Barthlott W, Mail M, Bhushan B, Koch K (2017). Nano-Micro Lett.

[54] Watson G S, Green D W, Cribb B W, Brown C L, Meritt C R, Tobin M J, Vongsvivut J, Sun M, Liang A-P, Watson J A (2017). ACS Appl Mater Interfaces.

[55] Barthlott W, Neinhuis C (1997). Planta.

[56] Barthlott W, Schimmel T, Wiersch S, Koch K, Brede M, Barczewski M, Walheim S, Weis A, Kaltenmaier A, Leder A (2010). Adv Mater (Weinheim, Ger).

[57] Tripathy A, Sen P, Su B, Briscoe W H (2017). Adv Colloid Interface Sci.

[58] Liu Q, Brookbank L, Ho A, Coffey J, Brennan A B, Jones C J (2020). PLoS One.

[59] Koc Y, de Mello A J, McHale G, Newton M I, Roach P, Shirtcliffe N J (2008). Lab Chip.

[60] Pernites R B, Santos C M, Maldonado M, Ponnapati R R, Rodrigues D F, Advincula R C (2012). Chem Mater.

[61] Stallard C P, McDonnell K A, Onayemi O D, O’Gara J P, Dowling D P (2012). Biointerphases.

[62] Zhu H, Guo Z, Liu W (2014). Chem Commun.

[63] Privett B J, Youn J, Hong S A, Lee J, Han J, Shin J H, Schoenfisch M H (2011). Langmuir.

[64] Crick C R, Ismail S, Pratten J, Parkin I P (2011). Thin Solid Films.

[65] Hizal F, Rungraeng N, Lee J, Jun S, Busscher H J, van der Mei H C, Choi C-H (2017). ACS Appl Mater Interfaces.

[66] Cai S, Wu C, Yang W, Liang W, Yu H, Liu L (2020). Nanotechnol Rev.

[67] Jeon S I, Lee J H, Andrade J D, De Gennes P G (1991). J Colloid Interface Sci.

[68] Ista L K, Mendez S, Lopez G P (2010). Biofouling.

[69] Bosker W T E, Patzsch K, Stuart M A C, Norde W (2007). Soft Matter.

[70] Haldar J, An D, Álvarez de Cienfuegos L, Chen J, Klibanov A M (2006). Proc Natl Acad Sci U S A.

[71] Wong S Y, Li Q, Veselinovic J, Kim B-S, Klibanov A M, Hammond P T (2010). Biomaterials.

[72] Buzzacchera I, Vorobii M, Kostina N Y, de los Santos Pereira A, Riedel T, Bruns M, Ogieglo W, Möller M, Wilson C J, Rodriguez-Emmenegger C (2017). Biomacromolecules.

[73] Kovacs B, Patko D, Klein A, Kakasi B, Saftics A, Kurunczi S, Vonderviszt F, Horvath R (2018). Sens Actuators, B.

[74] Liu X, Peng L, Meng J, Zhu Z, Han B, Wang S (2018). Nanoscale.

[75] Wang L-S, Gopalakrishnan S, Lee Y-W, Zhu J, Nonnenmann S S, Rotello V M (2018). Mater Horiz.

[76] Bullitt E, Makowski L (1995). Nature.

[77] Garrett T R, Bhakoo M, Zhang Z (2008). Prog Nat Sci.

[78] Asadi A, Razavi S, Talebi M, Gholami M (2019). Infection (Munich, Ger).

[79] Piatek R, Zalewska-Piatek B, Dzierzbicka K, Makowiec S, Pilipczuk J, Szemiako K, Cyranka-Czaja A, Wojciechowski M (2013). BMC Microbiol.

[80] Pinkner J S, Remaut H, Buelens F, Miller E, Åberg V, Pemberton N, Hedenström M, Larsson A, Seed P, Waksman G (2006). Proc Natl Acad Sci U S A.

[81] Eidam O, Dworkowski F S N, Glockshuber R, Grütter M G, Capitani G (2008). FEBS Lett.

[82] Soto G E, Hultgren S J (1999). J Bacteriol.

[83] Krachler A M, Ham H, Orth K (2011). Proc Natl Acad Sci U S A.

[84] Krachler A M, Mende K, Murray C, Orth K (2012). Virulence.

[85] Etayash H, Hancock R E W (2021). Pharmaceutics.

[86] Zasloff M (2002). Nature.

[87] Dürr U H N, Sudheendra U S, Ramamoorthy A (2006). Biochim Biophys Acta, Biomembr.

[88] Lai Y, Gallo R L (2009). Trends Immunol.

[89] Hancock R E W, Sahl H-G (2006). Nat Biotechnol.

[90] Lau Y E, Rozek A, Scott M G, Goosney D L, Davidson D J, Hancock R E W (2005). Infect Immun.

[91] Duplantier A J, van Hoek M L (2013). Front Immunol.

[92] Wimley W C, Hristova K (2011). J Membr Biol.

[93] Wang G, Li X, Wang Z (2016). Nucleic Acids Res.

[94] Overhage J, Campisano A, Bains M, Torfs E C W, Rehm B H A, Hancock R E W (2008). Infect Immun.

[95] de la Fuente-Núñez C, Korolik V, Bains M, Nguyen U, Breidenstein E B M, Horsman S, Lewenza S, Burrows L, Hancock R E W (2012). Antimicrob Agents Chemother.

[96] Lee J-K, Park Y (2020). Int J Mol Sci.

[97] Godballe T, Nilsson L L, Petersen P D, Jenssen H (2011). Chem Biol Drug Des.

[98] Bicker K L, Cobb S L (2020). Chem Commun.

[99] Beckloff N, Laube D, Castro T, Furgang D, Park S, Perlin D, Clements D, Tang H, Scott R W, Tew G N (2007). Antimicrob Agents Chemother.

[100] Tagliabue A, Rappuoli R (2018). Front Immunol.

[101] Bloom D E, Cadarette D (2019). Front Immunol.

[102] He T, Shi Z L, Fang N, Neoh K G, Kang E T, Chan V (2009). Biomaterials.

[103] He T, Zhang Y, Lai A C K, Chan V (2015). Biomed Mater.

[104] Altman G H, Diaz F, Jakuba C, Calabro T, Horan R L, Chen J, Lu H, Richmond J, Kaplan D L (2003). Biomaterials.

[105] Salehi S, Koeck K, Scheibel T (2020). Molecules.

[106] Aigner T B, DeSimone E, Scheibel T (2018). Adv Mater (Weinheim, Ger).

[107] Kiseleva A P, Krivoshapkin P V, Krivoshapkina E F (2020). Front Chem (Lausanne, Switz).

[108] Scheibel T, Zahn H, Krasowski A (2016). Silk. Ullmann's Encyclopedia of Industrial Chemistry.

[109] Herold H M, Scheibel T (2017). Z Naturforsch, C: J Biosci.

[110] Sehnal F, Sutherland T (2008). Prion.

[111] Garb J E, Ayoub N A, Hayashi C Y (2010). BMC Evol Biol.

[112] Sutherland T D, Young J H, Weisman S, Hayashi C Y, Merritt D J (2010). Annu Rev Entomol.

[113] Andersson M, Johansson J, Rising A (2016). Int J Mol Sci.

[114] Saric M, Scheibel T (2019). Curr Opin Biotechnol.

[115] Inoue S, Tanaka K, Arisaka F, Kimura S, Ohtomo K, Mizuno S (2000). J Biol Chem.

[116] Sehadova H, Zavodska R, Rouhova L, Zurovec M, Sauman I (2021). Int J Mol Sci.

[117] Kucerova L, Zurovec M, Kludkiewicz B, Hradilova M, Strnad H, Sehnal F (2019). Sci Rep.

[118] Eisoldt L, Smith A, Scheibel T (2011). Mater Today.

[119] Eisoldt L, Thamm C, Scheibel T (2012). Biopolymers.

[120] Bittencourt D, Oliveira P F, Prosdocimi F, Rech E L (2012). Genet Mol Res.

[121] Thamm C, Scheibel T (2017). Biomacromolecules.

[122] Collin M A, Clarke T H, Ayoub N A, Hayashi C Y (2018). Int J Biol Macromol.

[123] Altman G H, Horan R L, Lu H H, Moreau J, Martin I, Richmond J C, Kaplan D L (2002). Biomaterials.

[124] Ribeiro M, de Moraes M A, Beppu M M, Garcia M P, Fernandes M H, Monteiro F J, Ferraz M P (2015). Eur Polym J.

[125] Kuhbier J W, Allmeling C, Reimers K, Hillmer A, Kasper C, Menger B, Brandes G, Guggenheim M, Vogt P M (2010). PLoS One.

[126] Wendt H, Hillmer A, Reimers K, Kuhbier J W, Schäfer-Nolte F, Allmeling C, Kasper C, Vogt P M (2011). PLoS One.

[127] Yang M, Shuai Y, Zhou G, Mandal N, Zhu L, Mao C (2014). ACS Appl Mater Interfaces.

[128] Pedregal-Cortés R, Toriz G, Delgado E, Pollack G H (2019). Biofouling.

[129] Chigama H, Yokoi T, Furuya M, Yokota K, Kanetaka H, Kawashita M (2020). J Mater Sci: Mater Med.

[130] Amaley A, Gawali A A, Akarte S R (2014). Indian J Arachnol.

[131] Wright S, Goodacre S L (2012). BMC Res Notes.

[132] Tahir H M, Zaheer A, Rasheed A, Mukhtar M K, Yaqoob R, Liaqat I, Ahsan M M (2018). Acta Zool Bulg.

[133] Tahir M, Sattar A, Qamar S, Mukhtar M, Liaqat I, Ali M, Zaheer A, Arshad N, Yaqoob R, Naseem S (2019). J Anim Plant Sci.

[134] Carl N K, Alexander E D, Taylor A S, Jessica A R, Jonathan N P (2015). J Arachnol.

[135] Makover V, Ronen Z, Lubin Y, Khalaila I (2019). J R Soc, Interface.

[136] Zhang S, Piorkowski D, Lin W-R, Lee Y-R, Liao C-P, Wang P-H, Tso I-M (2019). J Exp Biol.

[137] Kaur J, Rajkhowa R, Afrin T, Tsuzuki T, Wang X (2014). Biopolymers.

[138] Fruergaard S, Lund M B, Schramm A, Vosegaard T, Bilde T (2021). iScience.

[139] Whittall D R, Baker K V, Breitling R, Takano E (2021). Trends Biotechnol.

[140] Heidebrecht A, Scheibel T (2013). Adv Appl Microbiol.

[141] Hedhammar M, Rising A, Grip S, Martinez A S, Nordling K, Casals C, Stark M, Johansson J (2008). Biochemistry.

[142] Stark M, Grip S, Rising A, Hedhammar M, Engström W, Hjälm G, Johansson J (2007). Biomacromolecules.

[143] Huemmerich D, Helsen C W, Quedzuweit S, Oschmann J, Rudolph R, Scheibel T (2004). Biochemistry.

[144] Doblhofer E, Scheibel T (2015). J Pharm Sci.

[145] Kumari S, Lang G, DeSimone E, Spengler C, Trossmann V T, Lücker S, Hudel M, Jacobs K, Krämer N, Scheibel T (2020). Data Brief.

[146] Kumari S, Lang G, DeSimone E, Spengler C, Trossmann V T, Lücker S, Hudel M, Jacobs K, Krämer N, Scheibel T (2020). Mater Today.

[147] Rabotyagova O S, Cebe P, Kaplan D L (2009). Biomacromolecules.

[148] Wohlrab S, Müller S, Schmidt A, Neubauer S, Kessler H, Leal-Egaña A, Scheibel T (2012). Biomaterials.

[149] Neubauer V J, Scheibel T (2020). ACS Biomater Sci Eng.

[150] Esser T U, Trossmann V T, Lentz S, Engel F B, Scheibel T (2021). Mater Today Bio.

[151] Widhe M, Shalaly N D, Hedhammar M (2016). Biomaterials.

[152] Widhe M, Johansson U, Hillerdahl C-O, Hedhammar M (2013). Biomaterials.

[153] Nilebäck L, Hedin J, Widhe M, Floderus L S, Krona A, Bysell H, Hedhammar M (2017). Biomacromolecules.

[154] Johansson U, Ria M, Åvall K, Dekki Shalaly N, Zaitsev S V, Berggren P-O, Hedhammar M (2015). PLoS One.

[155] Saric M, Eisoldt L, Döring V, Scheibel T (2021). Adv Mater (Weinheim, Ger).

[156] Jansson R, Lau C H, Ishida T, Ramström M, Sandgren M, Hedhammar M (2016). Biotechnol J.

[157] Jansson R, Thatikonda N, Lindberg D, Rising A, Johansson J, Nygren P-Å, Hedhammar M (2014). Biomacromolecules.

[158] Humenik M, Mohrand M, Scheibel T (2018). Bioconjugate Chem.

[159] Thatikonda N, Nilebäck L, Kempe A, Widhe M, Hedhammar M (2018). ACS Biomater Sci Eng.

[160] Thatikonda N, Delfani P, Jansson R, Petersson L, Lindberg D, Wingren C, Hedhammar M (2016). Biotechnol J.

[161] Leal-Egaña A, Lang G, Mauerer C, Wickinghoff J, Weber M, Geimer S, Scheibel T (2012). Adv Eng Mater.

[162] Petzold J, Aigner T B, Touska F, Zimmermann K, Scheibel T, Engel F B (2017). Adv Funct Mater.

[163] Borkner C B, Wohlrab S, Möller E, Lang G, Scheibel T (2017). ACS Biomater Sci Eng.

[164] Leal-Egaña A, Díaz-Cuenca A, Boccaccini A R (2013). Adv Mater (Weinheim, Ger).

[165] Blau A (2013). Curr Opin Colloid Interface Sci.

[166] De Rosa M, Carteni' M, Petillo O, Calarco A, Margarucci S, Rosso F, De Rosa A, Farina E, Grippo P, Peluso G (2004). J Cell Physiol.

[167] Widhe M, Bysell H, Nystedt S, Schenning I, Malmsten M, Johansson J, Rising A, Hedhammar M (2010). Biomaterials.

[168] Kramer J P M, Aigner T B, Petzold J, Roshanbinfar K, Scheibel T, Engel F B (2020). Sci Rep.

[169] Schacht K, Vogt J, Scheibel T (2016). ACS Biomater Sci Eng.

[170] Tasiopoulos C P, Gustafsson L, van der Wijngaart W, Hedhammar M (2021). ACS Biomater Sci Eng.

[171] Schacht K, Scheibel T (2011). Biomacromolecules.

[172] DeSimone E, Schacht K, Pellert A, Scheibel T (2017). Biofabrication.

[173] Neubauer V J, Trossmann V T, Jacobi S, Döbl A, Scheibel T (2021). Angew Chem, Int Ed.

[174] Schacht K, Jüngst T, Schweinlin M, Ewald A, Groll J, Scheibel T (2015). Angew Chem, Int Ed.

[175] Steiner D, Winkler S, Heltmann-Meyer S, Trossmann V T, Fey T, Scheibel T, Horch R E, Arkudas A (2021). Biofabrication.

[176] Shalaly N D, Ria M, Johansson U, Åvall K, Berggren P-O, Hedhammar M (2016). Biomaterials.

[177] Szymkowiak P, Tsiareshyna M, Koczura R (2020). Biologia (Cham, Switz).

[178] Alicea-Serrano A M, Bender K, Jurestovsky D (2020). J Arachnol.

[179] Harris T I, Gaztambide D A, Day B A, Brock C L, Ruben A L, Jones J A, Lewis R V (2016). Biomacromolecules.

[180] Gomes S C, Leonor I B, Mano J F, Reis R L, Kaplan D L (2011). Biomaterials.

[181] Liu F Y C (2021). bioRxiv.

[182] Nilebäck L, Chouhan D, Jansson R, Widhe M, Mandal B B, Hedhammar M (2017). ACS Appl Mater Interfaces.

[183] Chouhan D, Thatikonda N, Nilebäck L, Widhe M, Hedhammar M, Mandal B B (2018). ACS Appl Mater Interfaces.

[184] Huang T, Kumari S, Herold H, Bargel H, Aigner T B, Heath D E, O'Brien-Simpson N M, O'Connor A J, Scheibel T (2020). Int J Nanomed.

[185] Brennan S A, Ní Fhoghlú C, Devitt B M, O’Mahony F J, Brabazon D, Walsh A (2015). Bone Jt J.

[186] Li Y, Lin Z, Zhao M, Xu T, Wang C, Hua L, Wang H, Xia H, Zhu B (2016). ACS Appl Mater Interfaces.

[187] Seong M, Lee D G (2017). Curr Microbiol.

[188] Khalandi B, Asadi N, Milani M, Davaran S, Abadi A J N, Abasi E, Akbarzadeh A (2017). Drug Res (Stuttgart, Ger).

[189] Yu D, Kang G, Tian W, Lin L, Wang W (2015). Appl Surf Sci.

[190] Tang B, Li J, Hou X, Afrin T, Sun L, Wang X (2013). Ind Eng Chem Res.

[191] Tang B, Sun L, Kaur J, Yu Y, Wang X (2014). Dyes Pigm.

[192] Nadiger V G, Shukla S R (2016). J Text Inst.

[193] Doakhan S, Montazer M, Rashidi A, Moniri R, Moghadam M B (2013). Carbohydr Polym.

[194] He H, Cai R, Wang Y, Tao G, Guo P, Zuo H, Chen L, Liu X, Zhao P, Xia Q (2017). Int J Biol Macromol.

[195] Tao G, Cai R, Wang Y, Liu L, Zuo H, Zhao P, Umar A, Mao C, Xia Q, He H (2019). Mater Des.

[196] Currie H A, Deschaume O, Naik R R, Perry C C, Kaplan D L (2011). Adv Funct Mater.

[197] Khalid A, Bai D, Abraham A N, Jadhav A, Linklater D, Matusica A, Nguyen D, Murdoch B J, Zakhartchouk N, Dekiwadia C (2020). ACS Appl Mater Interfaces.

[198] Kumari S, Bargel H, Scheibel T (2020). Macromol Rapid Commun.

[199] Harvey D, Bardelang P, Goodacre S L, Cockayne A, Thomas N R (2017). Adv Mater (Weinheim, Ger).

[200] Seijsing F, Nilebäck L, Öhman O, Pasupuleti R, Ståhl C, Seijsing J, Hedhammar M (2020). MicrobiologyOpen.

[201] Zeplin P H, Berninger A-K, Maksimovikj N C, van Gelder P, Scheibel T, Walles H (2014). Handchir, Mikrochir, Plast Chir.

[202] Zeplin P H, Maksimovikj N C, Jordan M C, Nickel J, Lang G, Leimer A H, Römer L, Scheibel T (2014). Adv Funct Mater.

[203] Sommer C, Bargel H, Raßmann N, Scheibel T (2021). MRS Commun.

[204] Zhang Y, Zhou Z, Sun L, Liu Z, Xia X, Tao T H (2018). Adv Mater (Weinheim, Ger).

[205] Weiss A C G, Herold H M, Lentz S, Faria M, Besford Q A, Ang C-S, Caruso F, Scheibel T (2020). ACS Appl Mater Interfaces.

[206] Mulinti P, Diekjürgen D, Kurtzeborn K, Balasubramanian N, Stafslien S J, Grainger D W, Brooks A E (2022). Bioengineering.

[207] Zha R H, Delparastan P, Fink T D, Bauer J, Scheibel T, Messersmith P B (2019). Biomater Sci.

